# How Symbiotic Bacteria Survive Host Defenses

**DOI:** 10.1371/journal.pbio.1001164

**Published:** 2011-10-04

**Authors:** Robin Meadows

**Affiliations:** Freelance Science Writer, Fairfield, California, United States of America

**Figure:**
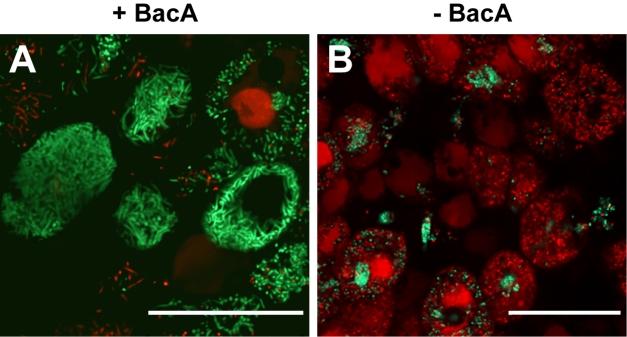
(A) BacA is critical for *Sinorhizobium meliloti* to form viable bacteroids (green stain) within plant cells. (B) In contrast, BacA-deficient mutant bacteria are rapidly killed (red stain).

There's a surprisingly fine line between bacterial symbiosis and chronic infection. While one is beneficial and the other detrimental, recent findings suggest that they share mechanisms for sidestepping host defenses. Plants in the pea family (legumes) have symbiotic nitrogen-fixing bacteria living in root nodule compartments that also contain antimicrobial compounds. For example, the nitrogen-fixer *Sinorhizobium meliloti* survives antimicrobial compounds called nodule-specific, cysteine-rich (NCR) peptides that are produced by alfalfa and related legumes. Intriguingly, *S. meliloti* is closely related to *Brucella abortus*, which causes abortions in cattle and can also cause debilitating chronic infections in people. Moreover, the host legume antimicrobial peptides that *S. meliloti* evades are similar to defensins, small proteins that help plants and animals kill bacteria.

Once inside root nodule compartments, *S*. *meliloti* differentiates from the free-living form into elongated bacteroids that fix nitrogen. This differentiation is mediated by antimicrobial NCR peptides on the plant side, but the corresponding factors on the bacterial side are unknown. An *S*. *meliloti* cytoplasmic membrane protein called BacA has long been known to be essential for bacteroid development in alfalfa relatives, but its role has remained uncertain. Recently, however, BacA has been linked to NCR peptides: while BacA is required for *S*. *meliloti* differentiation in legumes that produce NCR peptides (such as alfalfa and peas), this protein is not required for bacteroid development in legumes that lack these peptides (such as beans and lotus). Now, in this issue of *PLoS Biology*, Mergaert, Ferguson, and colleagues present compelling evidence that BacA is key to protecting *S*. *meliloti* from antimicrobial NCR peptides produced by host legumes.

To explore the role of BacA further, the researchers synthesized an antimicrobial peptide (NCR247) of the alfalfa relative *Medicago truncatula* and compared its effects on cultured *S*. *meliloti* with and without BacA. They found that while NCR247 decreases wild-type *S*. *meliloti* colony formation, this inhibition is more pronounced in a mutant strain that lacks BacA. In addition, this mutant's growth was restored by a plasmid carrying the *bacA* gene. Taken together, these findings suggest that BacA protects cultured *S*. *meliloti* against NCR peptides.

To test the effects of BacA in living plants, the researchers compared the viability of the wild-type and BacA-deficient *S*. *meliloti* strains. Nitrogen-fixing bacteria use infection threads to enter root nodule compartments, which contain NCR peptides that damage the cytoplasmic membrane of *S*. *meliloti*. Using a fluorescent dye test that stains dead bacteria red and stains living bacteria green, the researchers showed that both the wild-type and BacA-deficient *S*. *meliloti* were alive in the infection threads. In contrast, while the wild-type bacteria were still alive once inside the host root nodule compartments, most of the BacA-deficient mutants were dead. This suggests that without the protection of BacA, *S*. *meliloti* is rapidly killed by host antimicrobial NCR peptides in the compartments.

To confirm the hypothesis that BacA protects *S*. *meliloti* against antimicrobial NCR peptides, the researchers also applied the fluorescent dye test to an *M*. *truncatula* mutant that blocks NCR peptide transport into root nodule compartments. As expected, both wild-type and BacA-deficient *S*. *meliloti* thrived within this mutant's host compartments.

Extending the similarities between the symbiotic *S*. *meliloti* and bacteria that cause chronic infections, BacA-like proteins are also found in *B. abortus* and *Mycobacterium tuberculosis*. In addition, the *B. abortus* BacA is essential for chronic infections in mammals. Previous work had shown that *B. abortus* BacA protects *S. meliloti* mutants that lack this protein against antimicrobial peptides in alfalfa relatives, and the researchers likewise found that *B. abortus* BacA protected these *S. meliloti* mutants against the synthesized antimicrobial peptide (NCR247) in culture. Altogether, these findings suggest a functional similarity between the *S. meliloti* and *B. abortus* BacA proteins, and show that the latter protects against antimicrobial activity like that of the defensins found in its mammalian hosts.

This work suggests that by protecting *S. meliloti* against antimicrobial NCR peptides, BacA is key to establishing the symbiosis between these nitrogen-fixing bacteria and their legume hosts. How does BacA confer protection and limit the damage these compounds cause to the *S. meliloti* cytoplasmic membrane? Possibilities include a direct role for BacA in NCR transport or an indirect effect of BacA on outer membrane lipids. Because BacA is also key to establishing pathogenic bacteria including *B. abortus* in animal hosts, the answers could have widespread implications for understanding and ultimately treating such chronic infections in mammals.

**Haag AF, Baloban M, Sani M, Kerscher P, Pierre O, et al. (2011) Protection of** ***Sinorhizobium*** **Against Host Cysteine-Rich Antimicrobial Peptides Is Critical for Symbiosis doi:10.1371/journal.pbio.1001169**

